# Efficient Removal of Diclofenac from Aqueous Solution by Potassium Ferrate-Activated Porous Graphitic Biochar: Ambient Condition Influences and Adsorption Mechanism

**DOI:** 10.3390/ijerph17010291

**Published:** 2019-12-31

**Authors:** Nguyen Thi Minh Tam, Yunguo Liu, Hassan Bashir, Zhihong Yin, Yuan He, Xudong Zhou

**Affiliations:** 1 College of Environmental Science and Engineering, Hunan University, Changsha 410082, China; hnuliuyunguo@gmail.com (Y.L.); chohan_sawan@hotmail.com (H.B.); 2 Key Laboratory of Environmental Biology and Pollution Control (Hunan University), Ministry of Education, Changsha 410082, China; 3 School of Resource & Environmental Sciences, Hubei Key Laboratory of Biomass-Resources Chemistry and Environmental Biotechnology, Wuhan University, Wuhan 430079, China; yinzhihong@whu.edu.cn; 4 Center of Changsha Public Engineering Construction, Changsha 410013, China; heyuan1982@126.com (Y.H.); dongdonggod@sina.com (X.Z.)

**Keywords:** porous graphitic biochar, diclofenac sodium, potassium ferrate, water purification

## Abstract

Porous graphitic biochar was synthesized by one-step treatment biomass using potassium ferrate (K_2_FeO_4_) as activator for both carbonization and graphitization processes. The modified biochar (Fe@BC) was applied for the removal of diclofenac sodium (DCF) in an aqueous solution. The as-prepared material possesses a well-developed micro/mesoporous and graphitic structure, which can strengthen its adsorption capacity towards DCF. The experimental results indicated that the maximum adsorption capacity (q_max_) of Fe@BC for DCF obtained from Langmuir isotherm simulation was 123.45 mg·L^−1^ and it was a remarkable value of DCF adsorption in comparison with that of other biomass-based adsorbents previously reported. Thermodynamic quality and effect of ionic strength studies demonstrated that the adsorption was a endothermic process, and higher environmental temperatures may be more favorable for the uptake of DCF onto Fe@BC surface; however, the presence of NaCl in the solution slightly obstructed DCF adsorption. Adsorption capacity was found to be decreased with the increase of solution pH. Additionally, the possible mechanism of the DCF adsorption process on Fe@BC may involve chemical adsorption with the presence of H-bonding and π–π interaction. With high adsorption capacity and reusability, Fe@BC was found to be a promising absorbent for DCF removal from water as well as for water purification applications.

## 1. Introduction

Water pollution has become a serious environmental challenge globally due to the existence of various pollutants including pharmaceuticals, pesticides, and organic pollutants. Pharmaceuticals and pharmaceutically active compounds (PhACs) are considered to be emerging contaminants (ECs) because of their adverse effects on humans and living organisms [1]. Residual pharmaceuticals are released into water as a wastewater effluent, which poses serious threats to the environment. Numerous pharmaceuticals such as antibiotics, birth control pills, pain killers, and many other drugs enter into water, causing surface water [2], ground water [3], and marine water [4] pollution by daily consumption. Among PhACs, non-steroidal anti-inflammatory drugs (NSAIDs) are widely used over the world and detected in different environmental compartment at various concentrations [5]. Diclofenac (DCF) is considered the most commonly used NSAID in the world [6]. It was found as a salt of sodium or potassium in water systems, including surface water, drinking water, wastewater treatment plants effluent, and groundwater [7]. Large scale consumption of DCF globally increased its presence in wastewater and causes severe health concerns to living organisms [8]. Even with low concentrations in water, DCF still has a significant influence on human health [9]. Therefore, it is essential to remove DCF from water systems.

Effective wastewater treatment techniques are required to meet future discharge requirements. Numerous studies have investigated pharmaceutical removal from aqueous environments by reverse osmosis [10], photocatalysis [11], ozonation [12,13], and nanofiltration [14]. Unfortunately, these techniques require special expensive materials, costly energy, and chemical inputs. Therefore, an efficient, low-cost, and recyclable material for the removal of DCF from water is needed. Recently, adsorption has proven to be an efficient treatment technique for the removal of numerous ECs [15]. Adsorption technique has the advantages of easy operation, low cost, high efficiency, strong reproducibility and the availability of different adsorbents [16]. Biochar and its activated derivatives, as an attractive absorbent, have been widely employed in wastewater treatment and soil remediation [17]. It has been reported that biochar could remove toxic contaminants in wastewater such as pathogens [18], organic pollutants [19], and inorganic pollutants [20]. Biochar is obtained by heating organic biomass under a limited oxygen environment to obtain a material that has excellent adsorption capacity for various pollutants in wastewater due to a highly specific surface area and porous structure [21]. There are several methods for the modification of a biochar surface, including physical or chemical activation, steam activation, and coating for the contaminants’ removal from wastewater [22]. Among them, chemical activation has gained significant scientific attention. Activated biochar materials obtained by activated chemical have numerous advantages, such as having a wide spectrum of functional groups, highly specific surface area, and porous structure [23]. Those advantageous characteristics contribute to the increase in adsorption capacity of activated biochar. A variety of chemicals, including HNO_3_, ZnCl_2_, and KOH, were used as activators for converting biomass to activated biochar [24,25,26]. However, activated biochar has a relatively low electronic separated rate when compared with other materials such as graphene or carbon nanotubes [27]. Therefore, the magnetic properties of activated biochar should be improved to enhance its adsorption performance as well as reusability.

Recently, scientists have developed several methods of graphitization carbon biochar such as sacrificial template methods [28] or catalytic activation of biomass [29]. Potassium ferrate (K_2_FeO_4_) can efficiently oxidize organic compounds due to its strong oxidization [30]. It has been considered as an environmentally-friendly oxidant in both water and wastewater treatment [31]. However, there are fewer studies about the application of K_2_FeO_4_ in carbonization and graphitization of biochar at the same time. This work attempted to address this gap. Herein, we propose a one-step modification of biomass by using K_2_FeO_4_ as both activator (KOH) and catalyst (Fe). Pitch pine sawdust was utilized as the carbon source for the modification process. The obtained composite was known as Fe@BC. The characteristics of Fe@BC were examined by characterization techniques such as Scanning electron microscopy (SEM), Transmission electron microscopy (TEM), X-ray diffraction (XRD), Raman spectra, BET surface area measurement, Fourier-transform infrared spectrum (FTIR), X-ray photoelectron spectroscopy (XPS), and zeta potential. The as-prepared material was used to investigate adsorption performance toward the emerging contaminant of DCF. Adsorption capacity and mechanisms of DCF onto Fe@BC were investigated by batch adsorption experiments. The effects of adsorbent dosage, contact time, DCF initial concentration, and temperature on the adsorption process were also investigated in this work. In addition, the role of solution pH in the removal of DCF from aqueous solution by Fe@BC was discussed. Furthermore, absorbent recyclability was also investigated.

## 2. Materials and Methods

### 2.1. Chemical and Reagents

The pitch pine sawdust feedstock was obtained from Changsha (Hunan, China). Diclofenac sodium (DCF) (C_14_H_10_C_12_NNaO_2_) was purchased from Shanghai Yien Chemical Technique Co., Ltd (Shanghai, China). The chemical structure and examples of ecotoxicological effects of DCF are presented in Figure S1 and Table S1 (Supplement Materials), respectively. Potassium ferrate (K_2_FeO_4_) was purchased from Shanghai Macklin Biochemical Co., Ltd (Shanghai, China). All other analytical grade regents were used without purification. The ultra-pure water was prepared and used throughout the experiments by Millipore Milli-Q water purification system.

### 2.2. Preparation of Porous Graphitic Biochar

**Figure 1:**
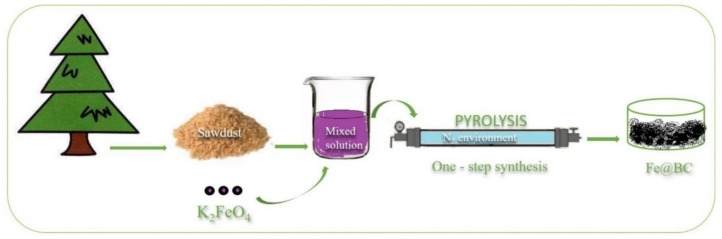
One-step synthesis of porous graphitic biochar.

The porous graphitic biochar was prepared from a simple chemical activation process by utilizing K_2_FeO_4_ as a chemical activator. Pitch pine sawdust, in a quantity of 3.0 g, was dispersed in 100 mL K_2_FeO_4_ aqueous solution (0.1 M) with continuous stirring for 8 h; the mixture was then dried overnight in an oven at 100 °C to obtain a solid compound. The mixture was then transferred into a tube furnace and heated at 900 °C for 2 h with a heating rate of 5 °C/min in N_2_ environment. The obtained porous graphitic biomass was thoroughly washed with 1 M HCl solution and ultra-pure water to remove any residual impurities. The sample was then dried at 80 °C in an oven overnight to obtain a solid compound, which was denoted as Fe@BC. The preparation process is schematically illustrated in Figure 1.

### 2.3. Characterization

The surface morphology of the Fe@BC was characterized by SEM (S4800, Hitachi, Japan) and the elemental composition of Fe@BC was observed by energy-dispersive X-ray spectroscopy (EDS) (La Mirada, CA, USA). The crystallinity of the as-prepared material was tested by XRD on a Bruker AXD D8 Advance diffractometer with Cu Kα source (acceleration voltage of 40 kV, 2 theta interval 0.02°, scan speed 0.1 s/step, λ = 0.15418 nm). Raman spectra were obtained from a LabRAM HR UV Raman spectrometer. The Brunauer-Emmett-Teller (BET) specific surface area of the samples was characterized by automatic surface analyzer (Quantachrome, EVO, USA). Magnetic characteristic of the as-prepared sample was measured with a vibrating sample magnetometer (VSM, Mpms (squid) XL-7, Quantum, CA, USA). For zeta potential analysis, 25 mg Fe@BC was dispersed in 25 mL ultra-pure water and the solution pH was adjusted in a range of 2–10 by adding negligible volumes of 0.1 NaOH or HCl. Zeta potential values were obtained from a zeta potential meter (Zetasizer Nano-ZS90, Malvern, UK). The XPS measurements were used to examine the binding energies of chemical composition (ESCALAB 250Xi, Thermo Fisher, USA) and FTIR was carried out to determine the surface functional groups of Fe@BC (IR Tracer-100, Shimadzu, Japan).

### 2.4. Adsorption Experiments

For adsorption experiments, the desired concentrations of the solution (20–100 mg·L^−1^) were prepared by dilution of stock solution (5 g L^−1^). Fe@BC was dried overnight at 100 °C in a vacuum oven, then used as adsorbent for the removal of DCF from water. To a 50 mL DCF solution, 50 mg of Fe@BC was added; then the mixture was shaken in an incubator shaker at constant speed of 160 rpm at room temperature. To obtain DCF concentration in solution after adsorption process, the mixture solution was filtered by the syringe filter (polytetrafluoroethylene -PTFE 0.45 µm). The residual concentrations of DCF were analyzed by UV-Vis spectroscopy (UV-2550, Shimadzu, Japan) at λ = 276 nm. All the measurements for the adsorption experiments were repeated thrice and average values were used.

The adsorption capacity (mg·g^−1^) of Fe@BC was obtained from the difference between the initial and the final concentrations of the DCF in the aqueous solution by the following equations:

(1) $$q_{e}\;=\frac{\left(C_{i}-C_{f}\right)V}{m}$$

(2) $$q_{t}=\frac{\left(C_{i}-C_{t}\right)V}{m}$$

(3) $$R\;=\;\frac{\left(C_{i}-C_{f}\right)\;}{C_{i}}\times \;100$$

where q_e_ is adsorption capacity at equilibrium point, q_t_ is adsorption capacity at time t (mg·g^−1^), R is the DCF removal efficiency rate (%), *C_i_* is the initial concentration of adsorbate (mg·L^−1^), *C_f_* is the final concentration after adsorption (mg·L^−1^), *C_t_* is the concentration of adsorbate solution at time t, *V* is the volume of the solution subjected to a single adsorption (L), and *m* is the mass of adsorbent taken during single adsorption (g).

### 2.5. Regeneration Experiments

After using the first-time adsorption DCF in solution (20 mg·L^−1^), the Fe@BC was rinsed with 25 mL 4 wt% NaOH solution at room temperature. The Fe@BC particles were transferred into a glass vial containing 50 mL pure acetone. The vial then was sealed by aluminum foil, agitated at 298 K (at 160 rpm speed) for 24 h. After desorption, Fe@BC was separated from the suspension solution by centrifugation. The whole process was repeated three times to obtain a sample, followed by drying at 353 K for reuse. In each cycle of adsorption test, 50 mg regenerated Fe@BC was added to 50 mL (20 mg·L^−1^) DCF, and the mixed solution was shaken at 298 K for 24 h with pH 6.5. The regenerated Fe@BC was used again for DCF adsorption under the same experimental conditions to check the reusability of the material. The whole process of regeneration and reusability was repeated five times.

## 3. Results

### 3.1. Material Characterization

**Figure 2:**
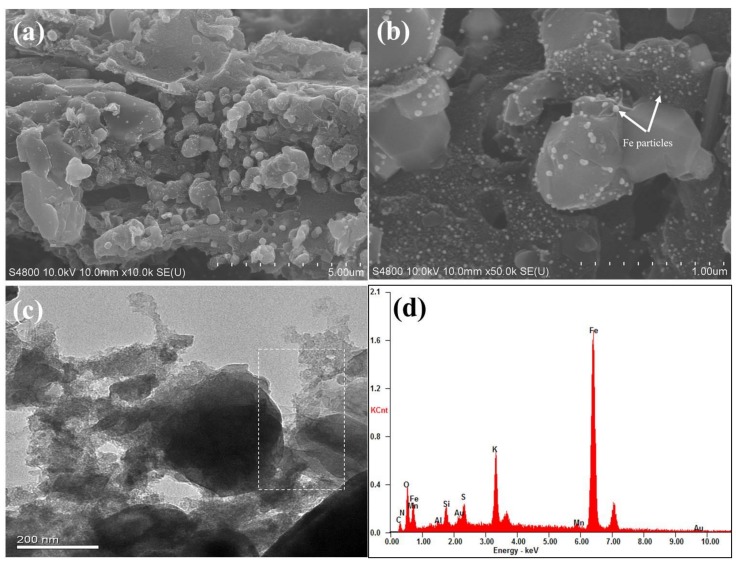
Scanning electron microscopy (SEM) images of (**a**) Fe@BC low magnification (**b**) Fe@BC high magnification (**c**) Transmission electron microscopy (TEM) image of Fe@BC (**d**) Energy dispersive spectra (EDS) of Fe@BC.

The surface morphology and microstructure of as-prepared sample were characterized by SEM and TEM, respectively. Figure 2a,b show the representative SEM images of Fe@BC in the different magnification conditions. After modification, the surface of Fe@BC presented an inhomogeneous structure with high roughness and the occurrence of many lumps with different dimensions. The high magnification SEM image (Figure 2b) shows that solid particles were allocated on the surface of Fe@BC and confirmed that Fe particles were loaded onto the Fe@BC surface. Furthermore, TEM image of Fe@BC (Figure 2c) indicated the presence of graphitic structure. To investigate the composition of Fe@BC, energy dispersive (EDS) analysis was performed. Notably, the Fe element stands for 72.44% on total wt% of Fe@BC particles, which confirmed that the as-prepared material was successfully activated by K_2_FeO_4_ (Figure 2d).

For further understanding of the surface morphology as well as the composition of as-prepared sample after DCF adsorption, SEM image and EDS analysis are presented in Figure S2 (Supplementary Materials).

**Figure 3:**
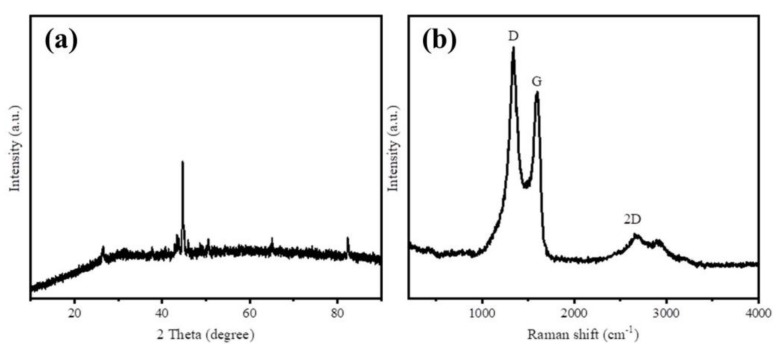
(**a**) X-ray diffraction (XRD) patterns and (**b**) Raman spectra of Fe@BC.

XRD and Raman spectra were employed to characterize the graphitization degree of as-prepared samples. The XRD patterns of Fe@BC in Figure 3a displays four diffraction peaks at 2θ values of 26.4° and 44.5°, which can be indexed to (002) and (001) planes that reflects the graphitic carbon (JCPDS No. 41-1487) [32]. These strong peaks revealed the crystalline graphitic structure on the surface of Fe@BC. The two obvious diffraction peaks at 65.1° and 82.4° indexed to (200) and (211) of Fe (JCPDS No. 06-0696) [33], respectively, indicate that the form of iron loaded on the Fe@BC surface was metallic Fe. In addition, Raman spectra was employed to investigate graphitic structure of the as-prepared sample and the results are presented in Figure 3b. Three prominent peaks were observed, which corresponded to D, G, and 2D bands, respectively. The D band at 1350 cm^−1^ corresponds to disordered and defective sites in carbon atoms; G band at 1570 cm^−1^ relates to the phonon mode in-plane vibration of sp^2^ bonded carbon atoms; and the 2D band at 2690 cm^−1^ corresponds to two phonon lattice vibration process, which indicated the typical representation of graphitic carbon [34,35]. Generally, the intensity ratio of D and G bands (I_D_/I_G_) was used to determine the order or disorder degree (crystallization) of carbonaceous materials. The high value (1.07) of I_D_/I_G_ ratio for the Fe@BC can be explained by the formation of pores that increased structural disorders during carbonization [36]. Raman spectrum of Fe@BC exhibited narrow D and G bands, accompanied with 2D band, which confirms the Fe@BC structure was successfully converted from an amorphous carbon structure into a graphitic carbon structure. In general, XRD and Raman results confirmed that carbonization and graphitization simultaneously took place when the activating process was conducted. After activation, the as-prepared sample possessed a high graphitization degree, which may improve its ability to remove organic pollutants in an aqueous solution.

**Figure 4:**
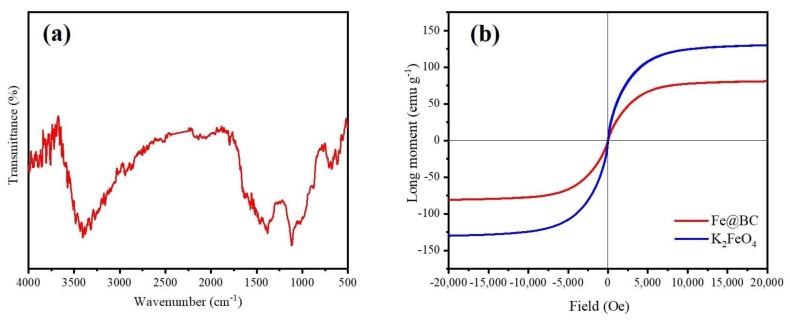
(**a**) Fourier-transform infrared spectrum (FTIR) spectra and (**b**) Magnetic hysteresis loop of Fe@BC and K_2_FeO_4._

The infra-red (FTIR) spectra was employed to identify the nature of functional groups on the surface of Fe@BC and the result is illustrated in Figure 4a. The spectra of Fe@BC showed stretching vibrations of O–H groups at 3405 cm^−1^ [37,38]. The peaks at 1390 cm^−1^ can be assigned to C=O axial deformation and presence of carboxyl groups [39]. The pronounced peak at 1115 cm^−1^ can be attributed to stretching vibrations of C–OH from phenol and hydroxyl groups [40]. Finally, two peaks at 675 and 615 cm^−1^ can be attributed to the stretching vibration of Fe–O [41]. The results revealed that Fe particles were deposited onto the surface of Fe@BC.

The magnetization hysteresis curve of Fe@BC was obtained using VSM at room temperature and the result is displayed in Figure 4b. Specific pertinent data showed the ferromagnetic characteristic of Fe@BC with saturation magnetization (M_s_) value of 80.9375 emu g^−1^, which confirmed that the as-prepared sample was magnetic enough to realize solid-liquid separation by using a permanent magnet. The values of remanence (M_r_), coercivity (H_c_) and the ratio of remanence and saturation magnetization (M_r_/M_s_) were 18.3694, 0.856, and 0.226, respectively. On the basis of the results, it is possible to conclude that Fe@BC possesses superparamagnetic characteristic because its ratio of M_r_/M_s_ was found to be less than 25% [42]. This property may enhance the as-prepared sample reusability because it could be easy to separate it from the treatment solution by magnetization process.

**Figure 5:**
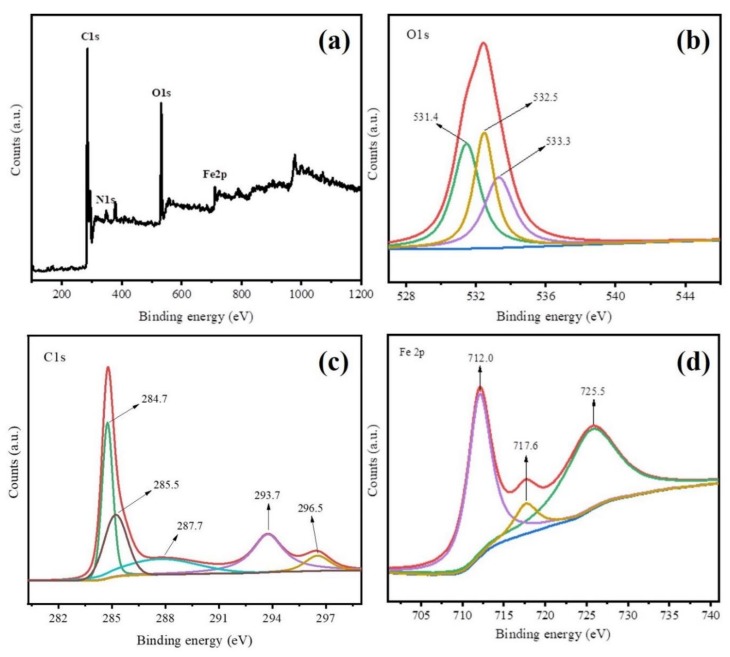
X-ray photoelectron spectroscopy (XPS) spectra of Fe@BC (**a**) XPS survey of, (**b**) O 1 s, (**c**) C 1 s, and (**d**) Fe 2p.

The XPS analysis was employed to investigate chemical composition and crystalline states of Fe@BC, and results are displayed in Figure 5. The XPS survey spectra results (Figure 5a) further demonstrated the presence of C, O, and Fe elements on the surface of as-prepared sample. These results were consistent with EDS analysis. Figure 5b shows the XPS spectrum of O 1 s for Fe@BC, where three peaks cantering at 531.4 eV, 532.5 eV, and 533.3 eV were clearly observed. The peak at 531.4 eV was attributed to signal of Fe–O (presence of lattice oxide oxygen in the oxides) [43], the peak at 532.5 eV suggested the presence of C=O, C–OH, or C–O–C groups [44], and the peak at 533.3 eV was attributed to oxygen in water molecules [45]. As shown in Figure 5c, C 1 s spectrum of the as-prepared sample shows five peaks, where the main sharp peak at 284.7 eV corresponded to C–C bonds with sp^3^ hybridization [46], and other four peaks centered at 285.5, 287.7, 293.7, and 296.5 eV were attributed to C=N, C–O, C=O, O=C–O bonds, respectively [47,48]. Furthermore, Fe 2p spectrum shows three peaks cantered at 712.0, 716.6, and 725.5 eV (Figure 5d). The peak at 712.0 eV was attributed to Fe 2p_3/2_ while the peak at 716.6 eV was attributed to Fe 2p_1/2_, which suggested that Fe^3+^ species presented on the surface of Fe@BC [49], and the peak centered at 725.5 eV was ascribed to Fe^0^ and Fe^2+^ [50].

**Figure 6:**
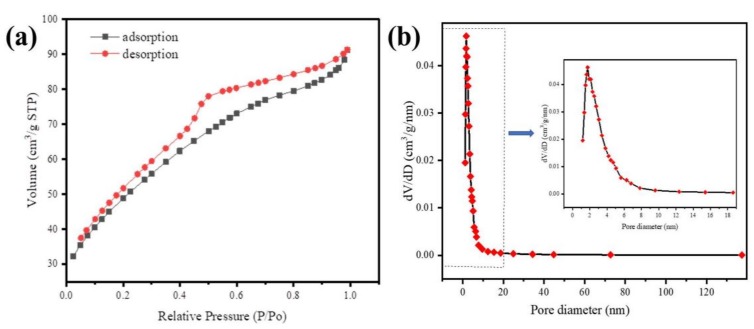
(**a**) N_2_ adsorption-desorption isotherms of Fe@BC and (**b**) Barrett-Joyner-Halenda (BJH) curve (inset pore-size distribution).

The N_2_ adsorption/desorption isotherms and mesoporous size distribution of Fe@BC are presented in Figure 6. The obtained N_2_ adsorption/desorption isotherm of as-prepared sample was identified as type IV with a hysteresis loop based on classification of International Union of Pure and Applied Chemistry (IUPAC) [51], which demonstrated the existence of mesoporous structure of adsorbent [52]. The specific surface area for the Fe@BC was approximately 175.8 m^2^ g^−1^. The Barrett-Joyner-Halenda (BJH) pore-size distribution plot is presented in Figure 6b. As can be seen, the pore-size distribution of Fe@BC appeared in a wide range (1.2–137.3 nm) with a value of 0.141 cm^3^ g^−1^ of total pore volume, and the average pore diameter was around 3.214 nm. The presence of mesopores on the Fe@BC would be beneficial for adsorption purposes in terms of accessibility of the adsorbent to the active sites for adsorption reactions. Additionally, N_2_ adsorption/desorption isotherms and mesoporous-sized distribution of as-prepared sample after DCF adsorption are illustrated as Figure S3 (Supplementary Materials).

The above material characterization results and discussions confirmed the pitch pine sawdust biomass was successfully converted to the porous graphitic structure. Fe@BC was synthesized by one-step activated using K_2_FeO_4_ as activator.

### 3.2. Diclofenac Sodium (DCF) Adsorption and Ambient Condition Influences

#### 3.2.1. The Effect of Absorbent Dosage

**Figure 7:**
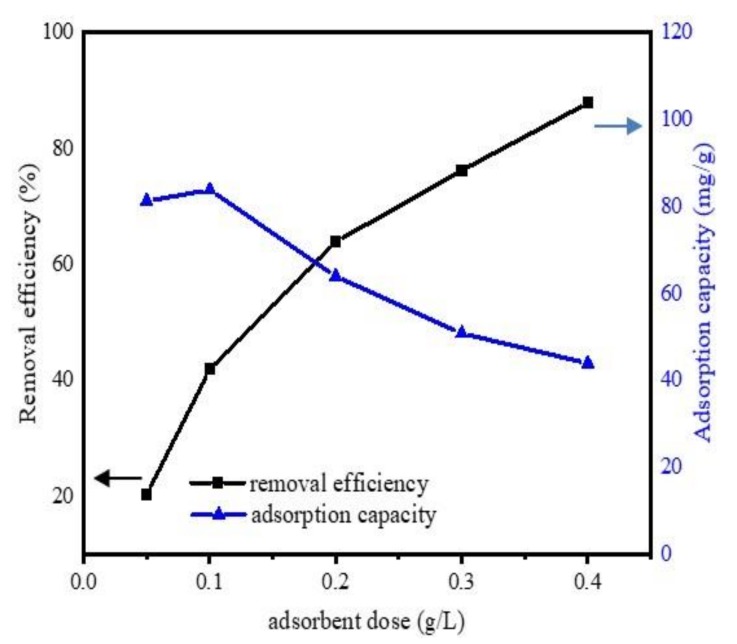
Effects of adsorbent dosage on the uptake of DCF onto Fe@BC.

The effect of adsorbent amount was determined by set of experiments, with a different adsorbent amount (0.05, 0.1, 0.2, 0.3, and 0.4 g L^−1^) and the experiment conditions were pH 6.5, DCF initial concentration 20 mg·L^−1^, temperature 298 K, contact time 24 h. The changes in adsorption efficiency and capacity of Fe@BC as a function of adsorbent amount are presented in Figure 7. It was observed that the removal efficiency was considerably enhanced by the increase of adsorbent dose. With a dosage of 0.05 g L^−1^, Fe@BC achieved a removal efficiency of 20.3%, and it increased from 45.2% to 87.8% with a dosage varying from 0.1 to 0.4 g L^−1^. However, the adsorption capacity decreased with the increase of adsorption dose. This observation can be attributed to the partial aggregation of biochar at higher concentrations, which might decrease the active sites on the surface of biochar and resulted to adsorption capacity decrease [53]. The removal efficiency increased with the increase of adsorption dose and achieved highest removal efficiency at the value of 0.4 g L^−1^ of adsorbent mass. The more the adsorbent dosage increased, the more active binding sites were promoted. These promoted active sites may participate in the DCF adsorption process.

**Figure 8:**
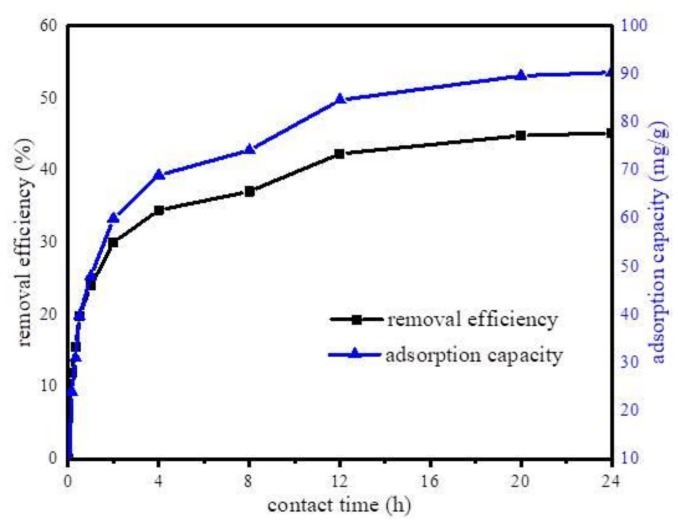
Effect of the adsorption time on the uptake of diclofenac sodium (DCF) onto Fe@BC.

Furthermore, the effect of contact time on the adsorption capacity of Fe@BC was determined. The experiments were performed at constant parameters (pH 6.5, adsorbent dosage 50 mg, DCF initial concentration 20 mg·L^−1^, temperature 298 K, contact time 24 h) at different time intervals. The effect of contact time is displayed in Figure 8. As can be observed, the adsorption capacity of DCF increased with the increase in contact time; the first 12 h adsorption was fast, then continued to increase slowly until it reached an equilibrium state at 24 h. The results indicated an increase in adsorption capacity and a decline in adsorption rate with time.

#### 3.2.2. Equilibrium Study and Kinetic Models

**Figure 9:**
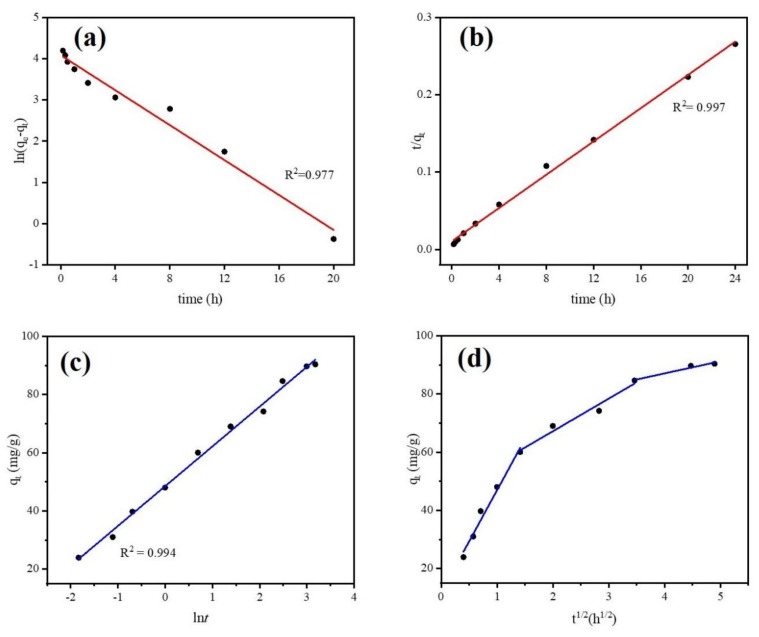
Kinetics study (**a**) Pseudo first-order model, (**b**) Pseudo second-order model, (**c**) Elovich model, (**d**) Intra-particle diffusion model.

Adsorption equilibrium study is a useful tool for understanding adsorption efficiency as well as adsorption mechanisms. It determines the equilibrium time of adsorbent microparticles. Adsorption kinetic models can predict potential rate limiting steps, chemical reactions, and the particle diffusion mechanism of DCF onto absorbent microparticles. However, biochar is known as an adsorbent that is not homogenous due to chemical reactions involved and because transport phenomena are often experimentally inseparable [54]. Thus, only applying elementary kinetic models (such as Pseudo first-order, Pseudo second-order) may not fully investigate the adsorption system of biochar. In this study, we employed Pseudo first-order, Pseudo second-order, and Elovich models to simulate the equilibrium experimental data. The model equations are given in Supplementary Materials Table S2. Figure 9 and Table 1 show the kinetic experimental results of different models for DCF adsorption onto Fe@BC and simulated parameters, respectively. From the applied kinetic models, linear regression coefficient (R^2^) is the basis for the accuracy of adsorption capacity prediction. As can be observed from Figure 9b and Table 1, the R^2^ values of 0.977, 0.997, and 0.994 were obtained from Pseudo first-order, Pseudo second-order, and Elovich kinetic models, respectively. Among them, a higher value correlation coefficient was obtained from the Pseudo second-order model and it is in good agreement with experimental data: the calculated value of q_e_ (92.5 mg·g^−1^) was near to experimental value (90.35 mg·g^−1^). Thus, fitting results suggested that adsorption rate of DCF correlated with chemical reaction/chemisorption [55]. In addition, Pseudo second-order proposed that the rate of adsorption for Fe@BC was affected by the availability of the sorption sites rather than the DCF solution concentration [56]. The Elovich model (Figure 9c) also presented a good fit for simulation of experimental data with a R^2^ value of 0.994, which confirmed the chemisorption was a dominant process during adsorption [57].

The intra-particle diffusion model was employed to further understand the mass transfer steps in DCF adsorption (Supplementary Materials Table S2). Multilinearity can be observed when different mechanisms with different rate constants are involved in sorption. In this situation, applying multiple linear regression is needed for each linear region [58]. Figure 9d and Table 2 show the simulated results of intra-particle diffusion model for DCF adsorption by Fe@BC. As can be seen, the plots of q_t_ versus t^1/2^ of Fe@BC were multi-linear, indicating three steps took place in the adsorption process. The first part (0–2 h), which shows a sharp curve, was attributed to the diffusion of DCF from the solution to the external surface of the adsorbent or the boundary layer diffusion of solute molecules [59]. The second part (2–12 h) indicated a gradual adsorption stage and intra-particle diffusion was the rate-limiting step. Finally, in the third part (12–24 h), q_t_ slightly changed, and the reaction reached an equilibrium state. Because the DCF particles were adsorbed onto Fe@BC surface, it led to a low DCF concentration left in the solution, and the intra-particle diffusion slowed down until it reached the saturation state. The sorption mechanism can be considered as intraparticle diffusion when the plot (q_t_ versus t^1/2^) is a straight line passing from the origin. In this case, the removal of DCF from aqueous solution was a complex process. It may be concluded that the diffusion process involved not only intra-particle diffusion but also boundary layer diffusion.

#### 3.2.3. Effect of DCF Initial Concentration

**Figure 10:**
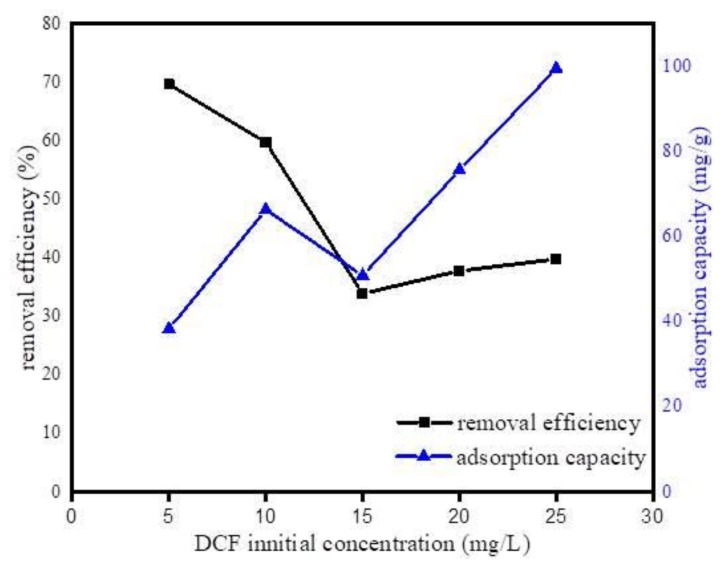
Effect of initial concentration of DCF on the uptake of DCF onto Fe@BC.

To investigate the initial concentration effect of DCF on the sorption process, adsorption isotherm experiment was performed with similar experimental conditions of the kinetic study accompanied with the initial concentrations of DCF varied from 5 to 25 mg·L^−1^. Figure 10 shows the variation of adsorption efficiency as a function of initial concentration. As can be observed, adsorption efficiency decreased with the increase in DCF initial concentration. This result happened because the higher initial concentration of DCF might cause the saturation of active binding sites on Fe@BC surface. However, it was observed that as the initial concentration of DCF increased the adsorption capacity also increased. This can be explained by the high initial concentrations of the DCF: as concentration gradient between bulk and solid liquid interface increased, this increased interface led to an upturn in the amount of adsorbed DCF on Fe@BC.

#### 3.2.4. Adsorption Isotherm

**Figure 11:**
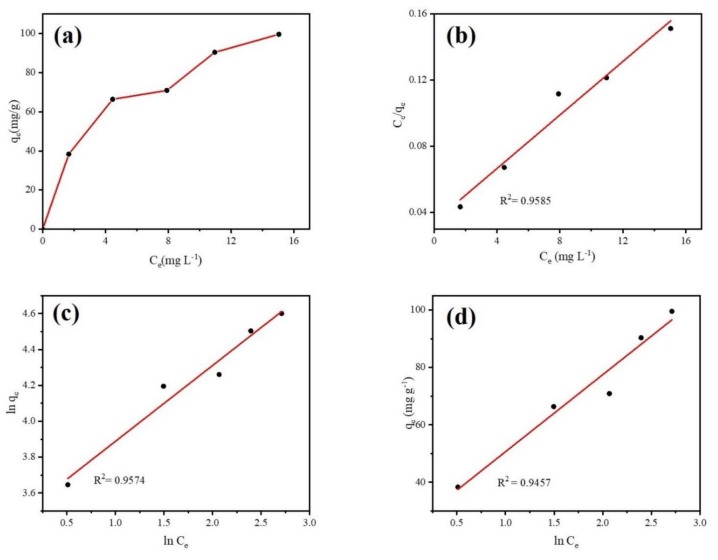
(**a**) Adsorption isotherms of DCF onto Fe@BC, (**b**) Langmuir, (**c**) Freundlich, and (**d**) Temkin plots for DCF adsorption over Fe@BC at 298 K.

Adsorption isotherm was conducted to explore the interactions between DCF and the Fe@BC microparticles as well as the adsorption mechanism. Equilibrium data were employed to study the adsorption isotherm models. Langmuir, Freundlich, and Temkin isotherm models were used to simulate the adsorption reactions. The isotherm model equations are given in  Supplementary Materials Table S3. Figure 11 illustrates the experimental adsorption isotherm of DCF on Fe@BC and the linear fitting curves of three isotherm models. As can be observed in Figure 11a, the adsorption isotherm of DCF over Fe@BC belonged to L-type isotherm, confirming the strong interaction between Fe@BC and DCF species [60].

Theoretically, different isotherm models can explain various mechanisms of the adsorption process. The Langmuir isotherm model describes quantitatively the formation of a monolayer adsorbate on the surface of adsorbent without any adsorbed specie interaction [61]. The Freundlich isotherm is commonly used to describe multilayer adsorption on the heterogeneous systems and is not restricted to the formation of the monolaye. The Temkin isotherm exhibits a brief interpretation of the interaction between adsorbent and adsorbate. It ignores the effect of low and higher values of concentration and assumes that the heat of adsorption of all the molecules in the layer would decrease linearly instead of being logarithmic with coverage [62]. The parameters obtained from three isotherm models are displayed in Table 3. It can be observed that Langmuir isotherm was more suitable when compared with the Freundlich isotherm and Temkin isotherm. The higher value of R^2^, which obtained from Langmuir model describes the homogenous adsorption process (uniform distribution of adsorbed molecules on the surface of Fe@BC due to monolayer adsorption). Furthermore, essential features of Langmuir isotherm to find out its suitability for adsorption process can be expressed as equilibrium parameter R_L_ (separation factor) [63]. The R_L_ is dimensionless constant and expressed in Equation (4):

(4) $$R_{L}\;=\;\frac{1}{1+(1+K_{L}C_{o})}$$

where C_o_ is the initial concentration in (mg·L^−1^) and K_L_ is in Langmuir equilibrium constant (L·mg^−1^).

From the Equation (4) above, calculated R_L_ value was 0.17, which was in the range (0–1) of favorable adsorption; this result further verified the suitability of the Langmuir isotherm model for intercepting the adsorption of DCF on Fe@BC.

#### 3.2.5. Thermodynamic Parameters

To understand detailed adsorption characteristics of DCF on Fe@BC, thermodynamic analysis was investigated. The thermodynamic parameters, including standard free-energy change (ΔG°), standard enthalpy change (ΔH°), and standard entropy change (ΔS°), were calculated from the different adsorption temperatures via the following equations and tabulated in Table 4.

ΔG° = −RTlnK_o_ (5)

ΔG° = ΔH° − TΔS°(6)

where K_o_ is the thermodynamic equilibrium constant calculated by plotting ln K_d_ (K_d_ = q_e_/C_e_) versus C_e_ and extrapolating C_e_ to zero, R = 8.314 (J mol^−1^ K^−1^) is universal gas constant and T (K) is the solution temperature in Kelvin. A linear plot between ΔG° versus T was performed to obtain slope and intercept, which could be assigned to ΔS° and ΔH°, respectively.

As seen in Table 4, the values of ΔG° were found to be negative at all temperatures, which indicated that the adsorption involved a favorable and spontaneous process. The adsorption capacity of 90.35 mg·g^−1^ at 298 K increased along with an increase in temperature to 99.5 mg·g^−1^ at 318 K. This small increase suggested that the effect of temperature on adsorption was insignificant. When environmental temperature increased, the surface coverage of the adsorbent tended to increase due to the expansion and creation of new active sites on Fe@BC. This may be the reason for the positive change of adsorption capacity when temperature increased. Additionally, 4.17 kJ mol^−1^ of ΔH° indicated the adsorption process was endothermic. The positive value of ΔS° suggested that randomness at the solid-liquid interface increased during the adsorption progress [57].

#### 3.2.6. Effect of Ionic Strength

**Figure 12:**
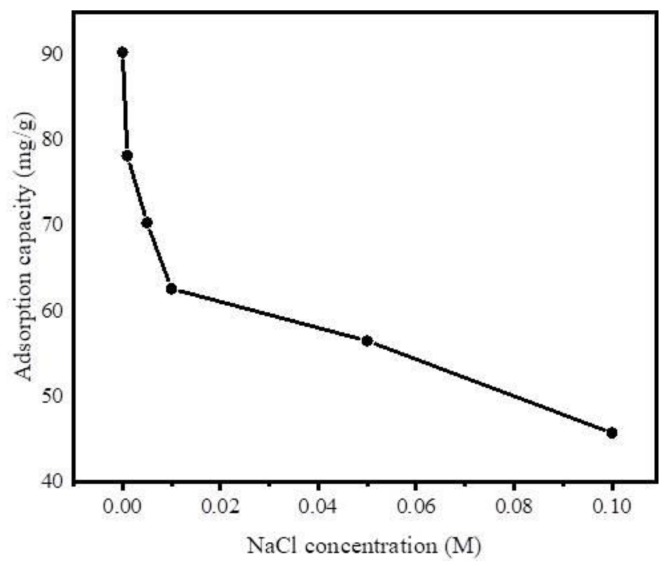
Effect of ionic strength on adsorption of DCF on Fe@BC.

Furthermore, to investigate the effect of ionic strength on adsorption of DCF on Fe@BC, different concentrations of NaCl (0.001 M–0.1 M) were added to the DCF solution, owing to the popular existence of this ionic compound in wastewater influent. As shown in Figure 12, the uptake of DCF by Fe@BC decreased with the increase of NaCl concentration. Theoretically, there are attractive electrostatic forces between the ions of adsorbate and adsorbent surfaces. Therefore, the adsorption capacity decreased with the increase of ionic strength. However, in a system with the existence of electrostatic repulsive, the adsorption capacity may show an increasing trend with the increase of ionic strength [64]. In addition, the infiltration of ions into the diffuse double layer surrounding Fe@BC surface may lead to the elimination of the repulsive interaction between the adsorbent surfaces, which could be unfavorable for the uptake of DCF onto Fe@BC.

#### 3.2.7. Comparison with Other Adsorbents

The comparison of adsorption performance between different reported carbon-based adsorbents towards DCF in terms of theoretical maximum adsorption capacity (q_max_) is presented in Table 5. It may be subjective when taking a comparison without considering material synthesis methods as well as reaction conditions; however, the comparison may support an overview in term of looking for the efficient adsorbents to remove DCF as well as other organic contaminants from water environment. As can be observed, Fe@BC presented a higher adsorption capacity for DCF when compared with other adsorbents. Thus, Fe@BC would be a potential adsorbent for organic contaminants adsorption due to its advantageous characteristics such as abundant origin materials, easy to synthesis (one-step activated), high porous graphitic structure, and copious surface functionalities.

### 3.3. Possible Adsorption Mechanism

Adsorption mechanism is important to understand the basics of adsorption process and surface area of a porous adsorbent. Therefore, the adsorption mechanism can be predicted by investigating the dependence of adsorption on pH value. The pH of the solution is a critical factor to understand the nature of an adsorbent surface and sorbate molecules as well as their mutual interaction [76,77]. Previous studies have reported that adsorption of pharmaceuticals contaminants on the surface of carbonaceous adsorbents was attributable to electrostatic interactions, H-bond interactions, π-π interactions, and van der Waals interactions. [78,79]. Furthermore, pH values less than pKa of DCF showed low solubility of DCF while the adsorption capacity increased inversely with the solution pH [80].

**Figure 13:**
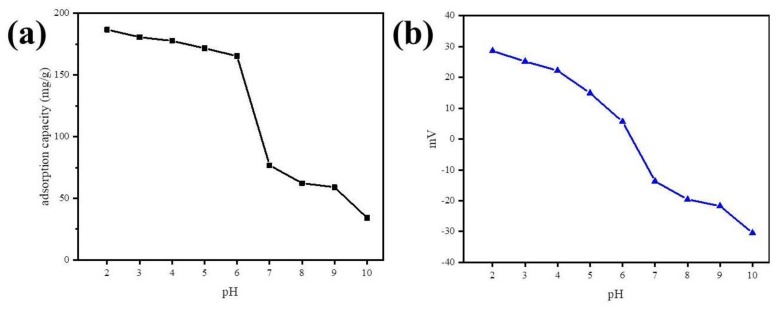
(**a**) Effect of solution pH on DCF adsorption by Fe@BC and (**b**) zeta potential of Fe@BC at different pH.

The impact of pH on the adsorption was investigated by varying the solution pH from 2.0 to 10.0 under experimental conditions, such as a kinetic study, and zeta potential of Fe@BC was measured at different pH values (Figure 13). Figure 13a shows the effect of pH on the removal process of DCF by Fe@BC. It was observed that there was not much decline in adsorption capacity with increase in pH from 2.0 to 6.5. The rapid decrease in adsorption capacity was seen when pH exceeded 6.5 and it continued to decrease with the increase of pH up to 10. Furthermore, the zeta potential of Fe@BC (Figure 13b) exhibited that surface charge decreased with the increase of pH of the solution, and point of zero charge (pH_pzc_) of Fe@BC was calculated (pH_pzc_ = 6.3). The pH_pzc_ value revealed the surface charges of as-prepared material (positive at <6.3 and negative at higher values).

The surface charge of DCF can be controlled by pKa (dissociation constant) value and it was a weak acid with pKa = 4.2 [57]. When the solution pH is higher than 4.2, it may possess a negative charge due to the ionization process. In contrast, when pH was lower than 4.2, DCF mostly presented in neutral form, and adsorbent surface may carry a positive charge. Therefore, the higher adsorption capacity at this pH value cannot be explained in terms of electrostatic attraction. Thus, other non-electrostatic adsorption mechanisms must be considered to explain the adsorption mechanism of DCF.

By considering the DCF molecules and polar functional groups of Fe@BC, hydrogen bonding (H-bonding) would be a possible mechanism for adsorption of DCF on Fe@BC; this interaction was widely employed to explain the elimination of emerging pollutants by various porous adsorbents [81,82]. To investigate the adsorption mechanisms, variation of DCF removal capacity as a function of wide pH range was considered as shown in Figure 13. The removal capacity would decrease drastically at pH > pKa of DCF by considering DCF to be an H-donor for H-bonding because, in this case, DCF molecules were deprotonated. Notably, the removal capacity was almost stable to pH ~ 6.5; accordingly, the DCF cannot be the H-donor. Therefore, Fe@BC would be an H-bond donor from the phenolic (pH ~ 10) and/or carboxylic (pH ~ 4.8) moieties presenting on its surface to the electronegative species in DCF (O-atoms as H-receptor).

**Figure 14:**
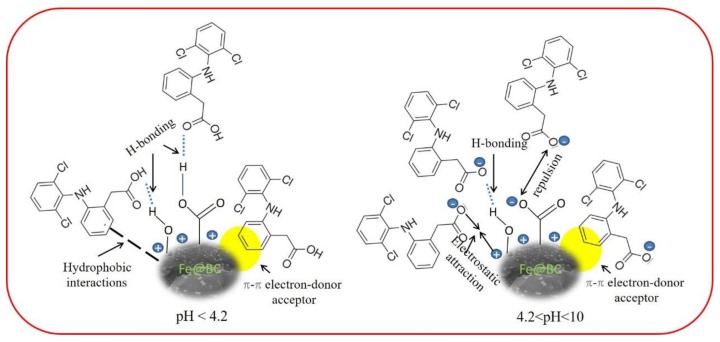
Proposed adsorption mechanism for DCF on Fe@BC at different pH values.

Given the DCF structure (Figure S1), the π–π electron donor–acceptor interaction between the aromatic rings of DCF (π-electron acceptor) and Fe@BC (π-electron donor) would occur [83]. The presence of chlorine atoms and carboxylic acid group in DCF could cause the π-electron density to decrease on its phenyl ring and facilitate π–π interactions with Fe@BC surface [71]. Lastly, hydrophobic interactions may occur by considering the hydrophobic characters of adsorbate surface and molecules [84]. Therefore, at the values higher than pKa of DCF and lower than pH_pzc_ of Fe@BC (4.2 < pH < 6.5), the electrostatic force of attraction would play an important role to adsorb DCF anions on the positive surface of Fe@BC. As can be seen in Figure 13a, DCF removal capacity was decreased with the increased in pH > 6.5, which can be explained by the electrostatic repulsive interaction between negatively charged adsorbent surface and the deprotonated carboxylic acid group. Nevertheless, as the pH increased to 10 or above, Fe@BC exhibited negligible adsorption ability because its surface posed a negative charge, which repelled DCF anions; accordingly, restricted the operation of H-bonding. It can be seen that the as-prepared sample showed considerable adsorption capacity (55.77 mg/g) at high pH values (pH ≥ 10) (Figure 13a), which can be attributed to π–π electron donor–acceptor [78,85]. On the basis of these results, the possible adsorption mechanisms of DCF onto Fe@BC at different pH values is shown in Figure 14.

### 3.4. Reusability and Regeneration

**Figure 15:**
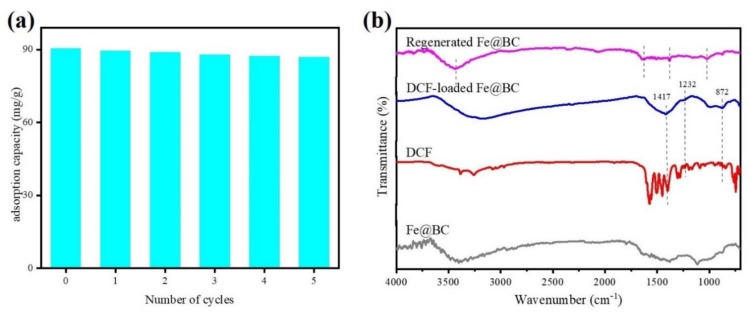
(**a**) Recyclability of Fe@BC for adsorption of DCF from aqueous solution and (**b**) FTIR spectra of Fe@BC, DCF, DCF-loaded Fe@BC, and regenerated Fe@BC.

To evaluate the reusability and cost effectiveness of the adsorbent, it is important to examine its performance through simulated desorption/regeneration cycles [52]. The FTIR analysis of Fe@BC, DCF, DCF-loaded Fe@BC, and recycled Fe@BC were compared and presented in Figure 15b. Stretching bands at 1417, 1232, and 872 cm^−1^ were observed for both DCF and DCF-loaded Fe@BC, verifying the adsorption of DCF onto Fe@BC. On the contrary, the vanishing of such bands and similar FTIR spectra (stretching bands at 3440, 1624, 1379, and 1024 cm^−1^) with fresh Fe@BC and a regenerated one suggested the successful removal of the adsorbed DCF, which is in accord with the results of the recycling tests (shown in Figure 15a). It was observed that DCF was successfully desorbed from the Fe@BC after 24 h of contact time using acetone as an eluent. As can be seen in Figure 15a, adsorption ability of DCF onto Fe@BC was 90.35 mg·g^−1^ at 298 K and pH 6.5 in the first cycle. However, after being reused in repeated cycles of adsorption/desorption, the adsorption capacity of the Fe@BC was expected to decrease. The loss of surface function groups during the desorption process may explain the reduction in the adsorbed amount of DCF by Fe@BC. As can be observed in Figure 15a, the adsorption ability of DCF onto the recycled FE@BC slightly reduced and remained at 86.94 mg·g^−1^ after five adsorption/desorption cycles. This implied that Fe@BC could be a potential and efficient adsorbent for DCF removal due to its considerable performance after five cycles of repeated usage.

## 4. Conclusions

Porous graphitic biomass carbon can be formed using pitch pine sawdust as a carbon source by one-step carbonization and graphitization process with K_2_FeO_4_ as chemical activation. The as-prepared sample (Fe@BC) was applied to remove diclofenac sodium (DCF) from aqueous solution. The Fe@BC presented a well-developed micro/mesoporous graphitic structure and Fe nano particles were observed on its surface. Via a study about the effects of contact time, it was found that adsorption of DCF by Fe@BC was fitted with Pseudo second-order model. In the adsorption equilibrium experiments, adsorption of DCF on Fe@BC Langmuir isotherm model was best fitted and suitable for the experiment data, indicating that the adsorption was a monolayer adsorption process. The Langmuir isotherm model showed higher adsorption monolayer capacity 123.45 mg·g^−1^ when compared with other carbon-based adsorbents. After five times recycling, the adsorption capacity of Fe@BC for DCF removal was only reduced by approximately 4%, which indicated a high stability and reusability of Fe@BC. Furthermore, the possible DCF adsorption mechanisms were also proposed. It was suggested that H-bonding and π–π interaction were possible adsorption mechanisms. Finally, Fe@BC can be considered as a potentially environmentally friendly adsorbent for the removal of DCF from water bodies due to its advantageous characteristics, such as facile preparation, high adsorption capacity, as well as recyclability.

## Figures and Tables

**Table 1:** Fitted parameters of different kinetic models for DCF adsorption on Fe@BC.

Pseudo First-Order			Pseudo Second-Order			Elovich		
k_1_ (min^−1^)	q_e,cal_ (mg·g^−1^)	R^2^	k_2_ (g·mg^−1^ min^−1^)	q_e,cal_ (mg·g^−1^)	R^2^	α (mg·g^−1^ min^−1^)	β (g·mg^−1^)	R^2^
0.212	59.34	0.977	0.011	92.5	0.997	473.89	0.0731	0.994

Experimental conditions: pH = 6.5, adsorbent dose = 50 mg, DCF initial concentration = 20 mg·L^−1^, V = 50 mL.

**Table 2:** Intra-particle diffusion model parameters of DCF uptake onto Fe@BC.

First Part			Second Part			Third Part		
K_id1_ (mg·g^−1^ min^−1/2^)	C_1_ (mg/g)	R^2^	K_id2_ (mg·g^−1^ min^−1/2^)	C_2_ (mg·g^−1^)	R^2^	K_id3_ (mg·g^−1^ min^−1/2^)	C_3_ (mg·g^−1^)	R^2^
35.17	11.72	0.978	11.19	44.78	0.968	4.18	70.31	0.966

Experimental conditions: pH = 6.5, adsorbent dose = 50 mg, DCF initial concentration = 20 mg·L^−1^, V = 50 mL.

**Table 3:** Adsorption isotherms parameters for DCF adsorption on Fe@BC.

Langmuir Isotherm			Freundlich Isotherm			Temkin Isotherm			
K_L_ (L·mg^−1^)	q_max_ (mg·g^−1^)	R^2^	n	K_F_ (mg·g^−1^)	R^2^	A_T_ (L·g^−1^)	b_T_	B (J·mol^−1^)	R^2^
0.236	123.45	0.9689	2.366	31.992	0.9681	2.429	92.326	26.835	0.945

Experimental conditions: pH = 6.5, adsorbent dose = 50 mg, DCF initial concentration = 20 mg·L^−1^, V = 50 mL.

**Table 4:** Thermodynamic parameters for adsorption of DCF on Fe@BC.

T (K)	lnK_o_	ΔG° (kJ mol^−1^)	ΔH° (kJ mol^−1^)	ΔS° (J K^−1^ mol^−1^)
298	1.14	−2.83	4.17	0.023
308	1.17	−3.01	-	-
318	1.25	−3.30	-	-

**Table 5:** DCF adsorption capacity of various adsorbents and Fe@BC.

Adsorbents	Adsorption Capacity q_max_ (mg·g^−1^)	References
Fe@BC	123.5	This study
Coca shell	63.5	[65]
Carbon xerogels	80.0	[66]
Activated carbon (Commercial)	76.0	[67]
Activated hydro chars from orange peels	52.2	[68]
Activated carbon	36.2	[69]
Activated carbon from olive-waste cakes	56.2	[70]
Granular carbon nanotubes	27.0	[71]
Tea waste derived activated carbon	62.5	[72]
Coconut shell activated carbon	103.0	[73]
Activated carbon from potato peel waste	68.5	[74]
Cyclamen persicum (herbaceous plant)	22.2	[75]

## Supplementary Materials

The following are available online at https://www.mdpi.com/1660-4601/17/1/291/s1, Figure S1: Structure of Diclofenac sodium, Figure S2: FT-IR spectra of Fe@BC, DCF-loaded Fe@BC and regenerated/recycled Fe@BC, Table S1: Kinetic models applied for the adsorption of DCF onto Fe@BC, Table S2: Isotherm models (linear form) applied for the adsorption of DCF onto Fe@BC.
