# Supplementary material for: Efficient Removal of Diclofenac from Aqueous Solution by Potassium Ferrate-Activated Porous Graphitic Biochar: Ambient Condition Influences and Adsorption Mechanism
Source: Int J Environ Res Public Health. 2019 Dec 31;17(1):291. doi: 10.3390/ijerph17010291 (PMC6981925; doi:10.3390/ijerph17010291)
Supplement: Supplementary file 1 [file ijerph-17-00291-s001.pdf]

Supplementary Material for:

Efficient removal of diclofenac from aqueous solution by potassium ferrate activated porous graphitic biochar: ambient condition influences and adsorption mechanism

Nguyen Thi Minh Tam [1,2] *, Yun-guo Liu [1,2], Hassan Bashir [1], Zhi-hong Yin[3]

[1] College of Environmental Science and Engineering, Hunan University, Changsha 410082, P. R. China.

[2] Key Laboratory of Environmental Biology and Pollution Control (Hunan University), Ministry of Education, Changsha 410082, P.R. China.

[3] School of Resource & Environmental Sciences, Hubei Key Laboratory of Biomass-Resources Chemistry and Environmental Biotechnology, Wuhan University, Wuhan, 430079, P.R. China.

* Corresponding authors at: College of Environmental Science and Engineering, Hunan University, Changsha 410082, P.R. China (N.T.M.Tam).

E-mail address: tamnm@hnu.edu.cn(N.T.M. Tam)

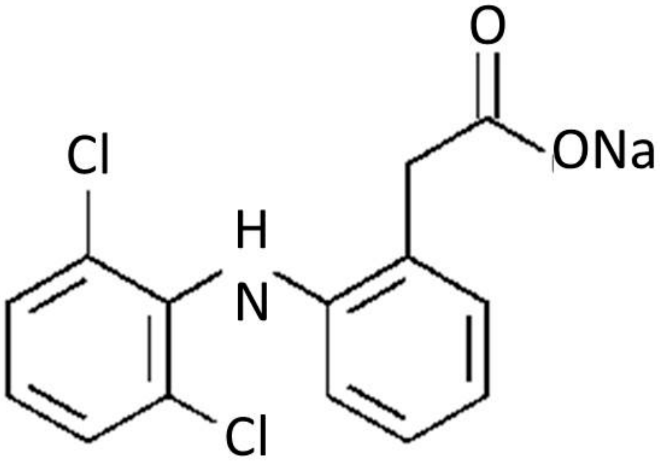

Fig. S1 Structure of Diclofenac sodium

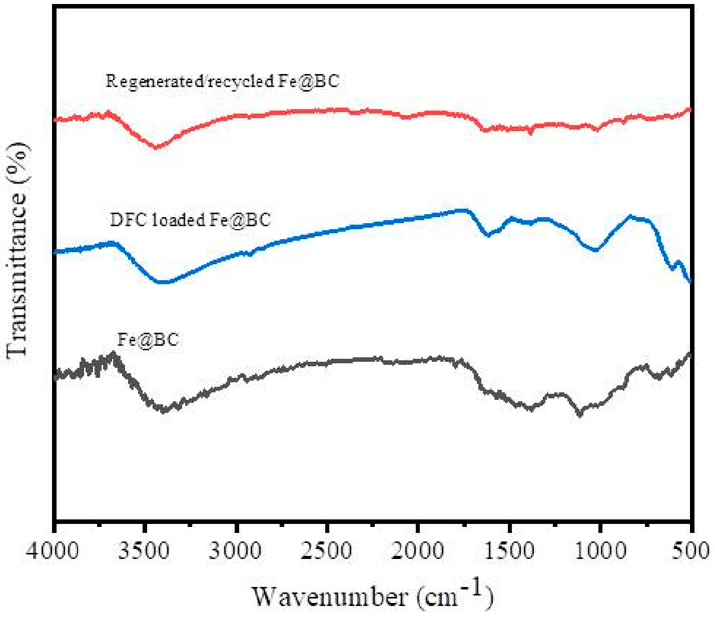

Fig. S2 FTIR spectra of Fe@BC, DFC loaded Fe@BC and regenerated/recycled Fe@BC

Table S1 Kinetic models applied for the adsorption of DCF onto Fe@BC

Model	Equation	Parameter	Interpretation
Pseudo first-order	$\ln (q_e - q_t) = \ln q_e - k_1 t$	q_e (mg g^{-1})	equilibrium adsorption capacity
		q_t (mg g^{-1})	adsorption capacity at time t
		k_1 (min^{-1})	adsorption rate constant
Pseudo second-order	$\frac{t}{q_t} = \frac{1}{k_2 q_e^2} + \frac{1}{q_e}$	q_t (mg g^{-1})	adsorption capacity at time t
		q_e (mg g^{-1})	equilibrium adsorption capacity
		k_2 (g mg^{-1} min^{-1})	adsorption rate constant
Intra-particle diffusion	$q_t = k_{id} t^{1/2} + C$	q_t (mg g^{-1})	adsorption capacity at time t
		k_{id} (mg g^{-1} $min^{-1/2}$)	intra-particle diffusion rate constant
		C (mg g^{-1})	intercept of the line
Elovich	$q_t = \frac{1}{\beta}\ln(\alpha\beta) + \frac{1}{\beta}\ln t$	q_t (mg g^{-1})	adsorption capacity at time t
		α (mg g^{-1} min^{-1})	initial sorption rate
		β (g mg^{-1})	desorption constant

Table S2 Isotherm models (linear form) applied for the adsorption of DCF onto Fe@BC

Model	Equation	Parameter	Interpretation
Langmuir isotherm	$\frac{C_e}{q_e} = \frac{1}{q_{max}K_L} + \frac{C_e}{q_{max}}$	C_e (mg L^{-1})	equilibrium concentration of the adsorbate
		q_e (mg g^{-1})	amount of adsorbed adsorbate per unit mass of adsorbent at equilibrium
		q_{max} (mg g^{-1})	maximum amount of adsorbed adsorbate for a complete monolayer
		K_L (L mg^{-1})	Langmuir equilibrium constant
Freundlich isotherm	$\ln q_e = \ln K_F + \frac{1}{n \ln C_e}$	C_e (mg L^{-1})	equilibrium concentration of the adsorbate
		q_e (mg g^{-1})	amount of adsorbed adsorbate per unit mass of adsorbent at equilibrium
		K_F (mg g^{-1})	Freundlich adsorption affinity coefficient
		n	adsorption intensity
Temkin isotherm	$q_e = B \ln A_T + B \ln C_e$<br>$B = \frac{RT}{b_T}$	q_e (mg g^{-1})	amount of adsorbed adsorbate per unit mass of adsorbent at equilibrium
		b_T	Temkin isotherm constant
		A_T (L g^{-1})	Temkin isotherm equilibrium biding constant
		R (J mol^{-1} K^{-1})	universal gas constant (8.314 J $mol^{-1}K^{-1}$)
		T (K)	temperature at 298 K
		B (J mol^{-1})	constant related to heat of sorption
